# Supplementary material for: Deletion of wfs1 Impairs Oligodendrocyte Precursor Cells Dorsal Distribution and Myelination Through the wfs1-hmgcs1 Axis in Zebrafish
Source: Biology (Basel). 2026 Aug 16;15(16):1402. doi: 10.3390/biology15161402 (PMC13510151; doi:10.3390/biology15161402)
Supplement: Supplementary file 1 [file biology-15-01402-s001.zip › File S1-Transcriptome sequencing data and differentially expressed genes (DEGs) in wfs1-- zebrafish at 5 dpf/2.KEGG/wfs1KOvsWT/UP/wfs1KOvsWT.up_KEGGbar.pdf]

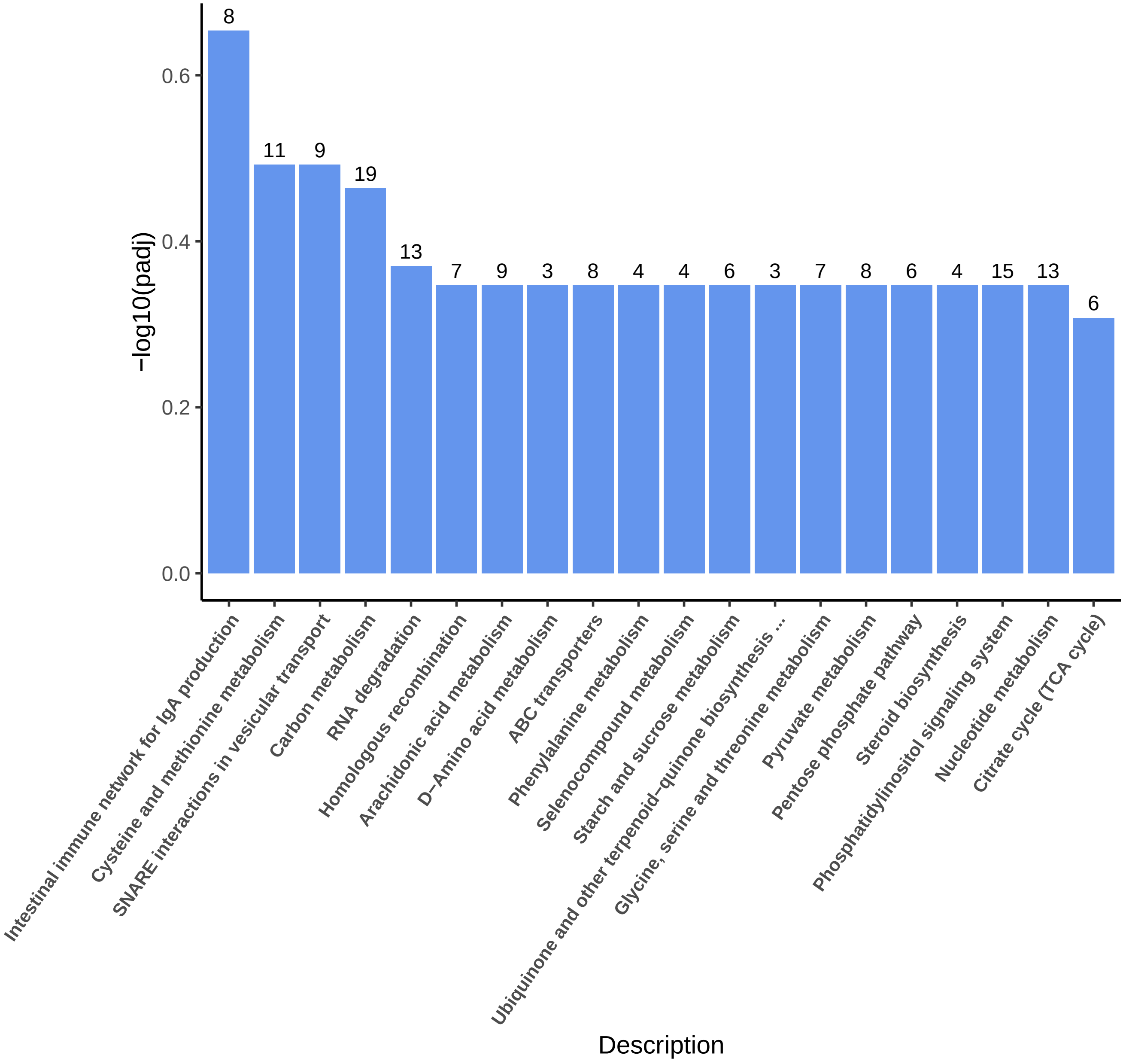

8
11
9
19
13
7
9
3
8
4
4
6
3
7
8
6
4
15
13
6
0.6
0.4
0.2
0.0
−log10(padj)
Intestinal immune network for IgA production
Cysteine and methionine metabolism
SNARE interactions in vesicular transport
Carbon metabolism
RNA degradation
Homologous recombination
Arachidonic acid metabolism
D−Amino acid metabolism
ABC transporters
Phenylalanine metabolism
Selenocompound metabolism
Starch and sucrose metabolism
Ubiquinone and other terpenoid−quinone biosynthesis ...
Glycine, serine and threonine metabolism
Pyruvate metabolism
Pentose phosphate pathway
Steroid biosynthesis
Phosphatidylinositol signaling system
Nucleotide metabolism
Citrate cycle (TCA cycle)
Description
